# Impact of oral health on nutritional status, self-perception of oral health and quality of life of institutionalized elderly

**DOI:** 10.4317/jced.57340

**Published:** 2021-02-01

**Authors:** Ilky-Pollansky Silva e Farias, Luiza-de Almeida-Souto Montenegro, Eduarda-Gomes-Onofre de Araújo, Maria-Leticia-Barbosa Raymundo, Arella-Cristina-Muniz Brito, Edson-Hilan-Gomes de Lucena, Simone-Alves de Sousa, Leopoldina-de Fátima-Dantas de Almeida, Yuri-Wanderley Cavalcanti

**Affiliations:** 1 Graduate Program in Dentistry. Master Student, School of Dentistry. Federal University of Paraíba, João Pessoa, PB, Brazil; 2 School of Dentistry. Federal University of Paraíba, João Pessoa, PB, Brazil; 3 Adjunct Professor, Department of Clinical and Social Dentistry, Federal University of Paraíba, João Pessoa, PB, Brazil

## Abstract

**Background:**

This study aimed to investigate the influence of oral health on nutritional status, self-perception of oral health and health related quality of life of institutionalized elders.

**Material and Methods:**

A cross-sectional study was conducted with 193 institutionalized elders living in the metropolitan region of João Pessoa (Brazil). The independent variables included were: 1) caries experience (DMFT index and its components); 2) use and need of dental prostheses; and 3) type of edentulism. The dependent variables included were related to nutritional status, self-perception of oral health and health-related quality of life. Data were submitted to a descriptive and comparative analysis, through correlation, association and difference tests, considering a significance level of 5% (*p*<0.05).

**Results:**

No statistical significant correlations or associations between the oral health status and nutritional status and quality of life were found (*p*>0.05). Individuals who did not need prosthesis had higher scores for nutritional status. Self-perception of oral health and health-related quality of life did not vary significantly according to the studied variables.

**Conclusions:**

The oral health status has a limited impact on the nutritional status, and does not impact the self-perception of oral health and quality of life of the institutionalized elders.

** Key words:**Nursing homes, oral health, quality of life.

## Introduction

A significant demographic transformation has been observed in the last decades, characterized by the increase in the proportion of the elders worldwide (1). In this context, the great challenge nowadays has been related to the elaboration of strategies to improve elders’ the quality of life, being the oral health status an important parameter for the general well being of these individuals (2).

Institutionalized elders have greater difficulty to access dental care compared to non-institutionalized, which predisposes the formers to a greater risk of deterioration of their oral health. The high cost of dental treatments, lack of knowledge related to oral care, mobility difficulties and the fear of dental interventions could be factors associated with lower access to dental care. Besides that, the lack of interest of dentists and dental practitioners in providing dental services may further aggravate the oral status of institutionalized elders (3).

Previous studies have shown that elderly people who live in nursing homes have an even worse state of oral health (4,5). Factors such as multi-morbidity, dependence to maintain one’s oral hygiene, compromised manual dexterity and excessive use of medications can interfere with the prevalence of oral diseases in institutionalized elders (6).

The evaluation of self-perception in oral health provides information on how individuals perceive their own oral health status with regards the functional, social and psychological dimensions, being those significantly associated with clinical findings and treatment needs (7). However, a previous study concluded that self-perception in oral health had minor influence on the oral health status of institutionalized elders, and the presence of pain is the main factor responsible for unfavorable self-perception (8). The identification of the factors associated with the poor oral health perception is needed for the elaboration of public policies directed to the improvement of health and quality of life, especially among the institutionalized elders (9).

There is evidence of an association between the presence of oral diseases and an increased risk of malnutrition (10). The high prevalence of tooth loss and inadequate oral rehabilitation are associated with loss of masticatory function and, consequently, with a greater intake of less nutritious and processed foods. In addition, residents of the nursing home are toothless, a condition that can result in limitations such as dietary restrictions, decreased pleasure in eating, unintentional weight loss and malnutrition (8).

Considering the growing population living in long-term institutions and the importance of oral health status in general health and quality of life of institutionalized elders, the aim of this study was to verify the impact of oral health on nutritional status, self-perception in oral health and health-related quality of life of institutionalized elders. In this study, the hypothesis was that the oral health influenced the nutritional status, the self-perception of oral health and the quality of life of the institutionalized elders.

## Material and Methods

-Ethical aspects

This study was in accordance with the ethical standards proposed by the national health council, as well as the Declaration of Helsinki (1964) and its subsequent modifications. The institutional ethical committee approved this study under protocol CAAE: 66122917.6.0000.5188. All participants signed a written and informed consent for information and research purposes.

-Subjects, design and location of the study

A cross-sectional study was conducted with 193 institutionalized elders living in the metropolitan region of João Pessoa (Paraíba, Brazil). This municipality is located in the Northeast region of Brazil and has 880,323 inhabitants, with a Human Development Index (HDI) of 0.763 and a Gross Domestic Product (GDP) per capita of R$23,169. A total of seven long-term institutions were identified in the metropolitan region of João Pessoa, all of them philanthropic. Most of the costs of these institutions are covered through the retirement income of the residents themselves.

Elders who were able to assimilate the proposed methodological instruments and who agreed to participate were included in the study. The participants answered a questionnaire and were then submitted to an oral examination by a group of previously calibrated researchers (Kappa ≥ 0.85).

-Instrument and variables

The independent variables analyzed in this study were: 1) dental caries experience (Decayed, Missed and Filled Teeth, DMFT according to the World Health Organization parameters); 2) use and need of dentures; and 3) type of edentulism (partial or total). Nutritional status, self-perception in oral health and health-related quality of life (HRQL) were considered as dependent variables in this study. Previously validated methodological instruments were used to collect the data.

-Nutritional status assessment

The Mini Nutritional Assessment - Short Form (MNA-SF) was used o evaluate the nutritional status of the participants. This instrument allows the identification of elderly individuals who are malnourished or at risk of malnutrition. The higher the score (maximum equal to 14), the better the nutritional status of the elders (11). Participants were classified into two categories, according to the reference parameters adopted to classify nutritional status: nourished (≥ 8 points) and malnourished (<8 points).

-Evaluation of self-perception in oral health

The self-perception in oral health of the elders was evaluated using the Geriatric Oral Health Assessment Index (GOHAI). This validated questionnaire is composed of 12 items that analyze self-perception in oral health in the following domains: physical function, psychosocial function and pain or discomfort. The participants answered the questions as follows: never (3 points), sometimes (2 points) and always (1 point)(12).

In this study, participants were included in two categories, according to the reference parameters adopted to classify the level of self-perception in oral health: good perception (≥ 34 points); and poor perception (<34 points).

-Assessment of health related quality of life (HRQL)

The SF-12 instrument was used to evaluate the health related quality of life related of institutionalized elders. The instrument consisted of 12 questions involving the following domains: physical, emotional, mental, pain, general health condition, vitality and social relations (13). Participants were classified into two categories, according to the median value for HRQL: poor HRQL (<62 points); and good HRQL (≥ 62 points).

-Statistical analysis

Data was tabulated and analyzed using program for this purpose (Statistical Package for Social Sciences software, IBM-SPSS, v.21, Chicago, IL). A descriptive analysis of the data was conducted to calculate the absolute and relative frequencies.

The median value of the Health Related Quality of Life (HRQL – median = 62) was considered as a reference for the dichotomization of this variable. For the other dependent variables included (nutritional status and self-perception of oral health), the reference values were obtained according to the score established by the questionnaires related to nutritional status (MNA threshold = 8 points) and self-perception in oral health (GOHAI threshold = 34 points).

 Statistical tests for correlation (Spearman), association (Chi-square or Fisher) and difference (Mann-Whitney or Kruskal-Wallis) were used to verify the relationship between the independent and dependent variables of this study, considering 5% level of significance.

## Results

The population of this study was majoritarian female (72%, n=139) and above 80 years (54.9%, n=106). No statistically significant correlation (Table 1) was observed between dental caries experience (DMFT index and its components) and the dependent variables (nutritional status, self-perception in oral health and HRQL). Also, there were no statistically significant associations between oral health, nutritional status, self-perception in oral health and HRQL (Table 2).

**Table 1:**
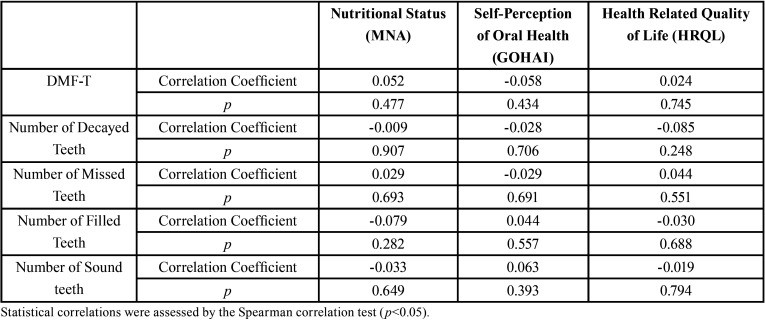
Correlation between caries experience (CPO-D index and its components) with scores obtained in the evaluation of the Nutritional Status, Self-perception in Oral Health and Health-Related Quality of Life of institutionalized elderly.

**Table 2:**
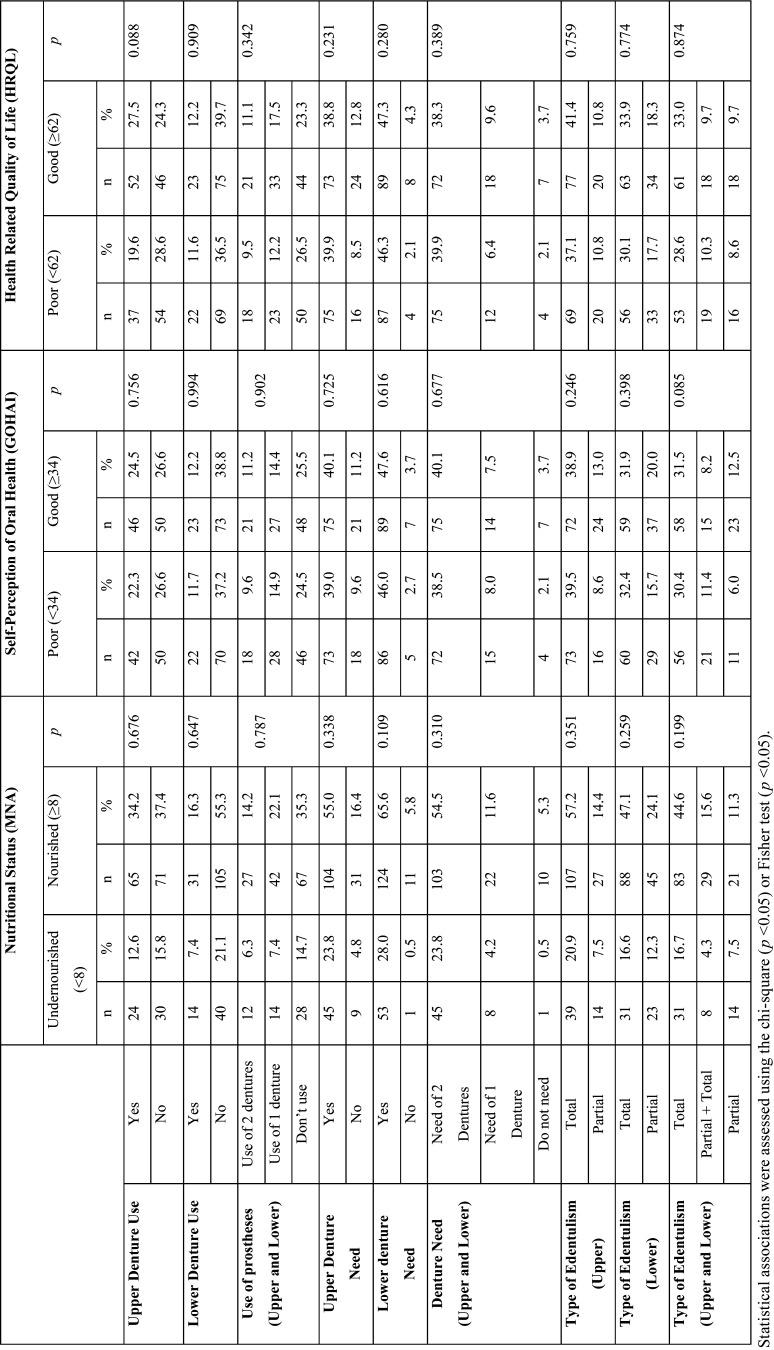
Nutritional status, self-perception in oral health and health-related quality of life of institutionalized elderly, according to the distribution of use and need of prosthesis and type of edentulism.

Individuals who do not need dentures presented higher scores for the nutritional status (*p*<0.05). Self-perception in oral health and HRQL did not vary significantly as a function of the studied variables (*p*>0,05) (Table 3). These findings show that oral health status has limited impact on nutritional status, self-perception in oral health and HRQL of institutionalized elders.

**Table 3:**
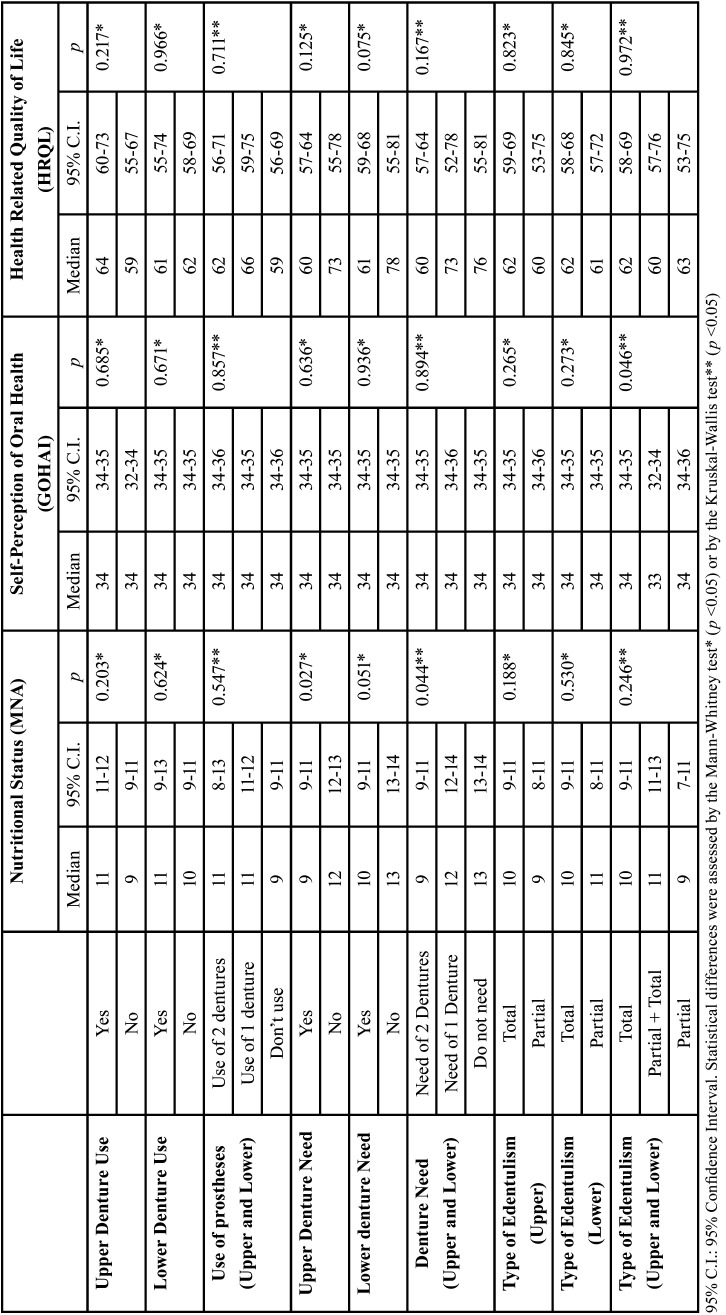
Median nutritional status, self-perception in oral health and health-related quality of life of institutionalized elderly, according to the use and need of prosthesis and type of edentulism.

Discussion 

The findings of this study show that, overall, oral health status did not interfere with the risk of malnutrition, poor self-perception in oral health, and poor health-related quality of life of institutionalized elders. Based on that, the null hypothesis was accepted.

Possibly, other health problems experienced by institutionalized elders with regards to psychological, physical and social domains would interfere more significantly with the dependent variables under study. Based on that, oral health would have a secondary role on the nutritional status and on HRQL. In addition, the poor relationship between oral status and self-perception on oral health suggests that institutionalized elders do not perceive appropriately their poor oral status, or do not give enough importance to it (14).

Although the National Oral Health Survey provides data referring to the elderly population in Brazil with an age range of 65-74 years, there is no data of population-based study on oral health status of Brazilian institutionalized elders (14). Previous reports show that institutionalized elders have poor oral health status, consisted of high DMFT index values and high prevalence of denture need (6,15). This scenario was confirmed in the present study. However, differently than expected for community-dwelling elders, oral heath presented a limited impact on elders’ health status and on how those individuals perceive their health.

In this study, the oral health condition did not influence the self-perception in oral health of the elderly participants. Even though oral health problems were prevalent among institutionalized older adults, their perception of oral health was positive (16). Possibly, these individuals had experienced poor oral health status during a long time and perceive the tooth loss as a natural process of their life-course. In addition, the events that resulted in institutionalization are probably not associated with the poor oral health status (17). It is also important to point out that other factors may influence the self-perception of the elders in oral health, such as social, cultural, age, sex and previous psychological experiences (18-20).

The poor oral health has been considered a risk factor for malnutrition, mainly in hospitals and nursing care homes (21). A previous study showed that negligence with oral hygiene could compromise the general health of the individual, interfering mainly in the nutritional status (22). In addition, biofilm accumulation and the presence of dental caries and periodontal diseases can negatively impact nutrient intake, contributing to the increase of malnutrition rates (23). However, the present study did not consider those parameters for the evaluation of oral health status.

The findings of this study revealed that 114 institutionalized elders were classified as total edentulous, a cause for alarming and concern. The absence of occluding pairs and lower masticatory function was therefore expected to perform a significant impact on nutritional status (24). However, elders living in long-term care institutions have a strict control of their diet and diary calories intake. Even though institutionalized elders have lower number of teeth or do not use dentures, they are not likely undernourished. In addition to the strict dietary control, institutionalized elders are probably used to intake hard food without enough occluding pairs, since they have experienced teeth loss for a long time. Nevertheless, other factors than oral health may interfere with nutritional status and health professional assistance is necessary during the aging process (25).

The institutions vary in relation to the minimum number of people destined to the elderly care, being rare the inclusion of the dental staff within the professional team. Although oral health is an important issue within the general health, caregivers have multiple concerns with regards to elderly care. Since most part of elders does not have natural teeth and elders have good perception of their own mouth, caregivers do not give enough importance oral health (26). None of the long-term care institutions visited during this study had appropriate dental care programs directed to institutionalized elders.

For a long time, dentistry was based on healing and mutilating practices, which did not value the preservation of teeth. It is important to consider that a significant portion of the elders investigated attribute little, if any, importance to the oral health. In part, these elderly people face health problems that go beyond the difficulty of chewing or shame when smiling. Theses findings are of relevance for better comprehension of oral health during elderly life, mainly for institutionalized people. The restriction to perform daily living activities and limited family-life may be much more involved in the poor quality of life of these elderly individuals. Besides that, low social interactions and high medication intake experienced in these institutions makes the aesthetic perception of the smile underestimated. Qualitative studies should be designed to better understand the psychological aspects involved in the quality of life of these individuals.

Although the sample is composed of 40% of the universe of participants, this study is representative for the elderly living in nursing care homes that have little or no cognitive impairment. These results can be applied to other institutions located in the northeast or institutionalized elders in other countries with similar socioeconomic and cultural conditions.

## Conclusions

Oral health status did not affect the risk of malnutrition, self-perception in oral health, and the quality of life of the institutionalized elders. This is of relevance because oral health status at the end of life does not impair health-related outcomes. Interventions on oral health among those individuals may be balanced with their potential benefits.
